# Immobilization of Eversa Lipases on Hydrophobic Supports for Ethanolysis of Sunflower Oil Solvent-Free

**DOI:** 10.1007/s12010-021-03774-8

**Published:** 2022-01-20

**Authors:** Daniela Remonatto, J. Vladimir Oliveira, J. Manuel Guisan, Débora Oliveira, Jorge Ninow, Gloria Fernandez-Lorente

**Affiliations:** 1 Department of Engineering of Bioprocesses and Biotechnology, School of Pharmaceutical Sciences, São Paulo State University (UNESP), 14800-903 Araraquara, SP Brazil; 2 Department of Chemical and Food Engineering, UFSC, 88040-900 Florianópolis, SC Brazil; 3 Departamento de Biocatálisis, Instituto de Catálisis-CSIC, UAM, Cantoblanco, 28049 Madrid, Spain; 4 Departamento de Biotecnología y Microbiología de los Alimentos, Instituto de Alimentación, CIAL (CSIC-UAM), Madrid, Spain

**Keywords:** Lipase, Eversa® Transform, Eversa® Transform 2.0, Solvent-free, Hydrophobic supports, Immobilization

## Abstract

Lipases are an important group of biocatalysts for many industrial applications. Two new commercial low-cost lipases Eversa® Transform and Eversa® Transform 2.0 was immobilized on four different hydrophobic supports: Lewatit-DVB, Purolite-DVB, Sepabeads-C18, and Purolite-C18. The performance of immobilized lipases was investigated in the transesterification of sunflower oil solvent-free in an anhydrous medium. Interesting results were obtained for both lipases and the four supports, but with Sepabeads support the lipases Eversa showed high catalytic activity. However, the more stable and efficient derivative was Eversa® Transform immobilized on Sepabeads C-18. A 98 wt% of ethyl ester of fatty acid (FAEE) was obtained, in 3 h at 40ºC, ethanol/sunflower oil molar ratio of 3:1 and a 10 wt% of the immobilized biocatalyst. After 6 reaction cycles, the immobilized biocatalyst preserved 70 wt% of activity. Both lipases immobilized in Sepabeads C-18 were highly active and stable in the presence of ethanol. The immobilization of Eversa Transform and Eversa Transform 2.0 in hydrophobic supports described in this study appears to be a promising alternative to the immobilization and application of these news lipases still unexplored.

## Introduction

Lipases (triacylglycerol acyl- hydrolase, EC 3.1.1.3) are biocatalysts naturally robust and efficient, can be used for the production of many different molecules, and have a wide range of industrial applications thanks to their broad specificity and commercial accessibility [1–3]. Among these molecules of interest is ethyl oleate, which is an ester with applications in cosmetics, in the food industry but also in the production of biofuels as biodiesel [4–6].

In December 2014, a new soluble lipase called Eversa® Transform was launched by Novozymes S / A (Denmark) with potential for use in industrial enzymatic biodiesel production processes [7]. After, in May 2016, the company announced the second generation of lipase, Eversa Transform 2.0, with greater thermostability [7].

Most of the published studies about Eversa lipases are transesterification of triglycerides or esterification of free fatty acids and used these enzymes in the free form [8–12]. Even though Eversa enzymes may be used in free form, their immobilization may enhance many properties such as stability and resistance to inhibitors chemicals [13]. Therefore, there are very few studies into the immobilization of Eversa lipases which is still a field to be better explored [13–15].

The lipases, as well as other enzymes, are inhibited by diverse components (temperature, several pH, etc.), their stability is moderate, besides, they are usually water-soluble, these properties, are a problem if they are going to be used as industrial biocatalysts, where they are expected to perform their function under standardized conditions [16]. Therefore, there is an urgent need to develop effective methods to improve enzymes’ structural stability and biological activity in practical applications [17]. Enzyme immobilization is a technique that allows the reuse of the biocatalyst and generally improves its stability, these characteristics are of fundamental importance to turn industrial biocatalysis into viable [18].

Lipases can be immobilized in various types of solid supports by different methods including, for example, adsorption and covalent attachment. Today, the most popular strategy of lipase immobilization is based on the physical adsorption on hydrophobic supports, which under mild conditions (low ionic strength and neutral pH) [3,15, 19–21].

Most lipolytic enzymes have a specific mechanism concerning activation, its active site is cover by a lid-like (polypeptide chain) which render the active site inaccessible, however, when a lipase was bound to a lipid interface, a conformational change took place causing the lid to move away whereby the active site of the lipase became fully accessible [22–24]. When the lipases come into contact with strongly hydrophobic solid surfaces the reaction in a way that is similar to the way they recognize natural substrates and therefore undergoes interfacial activation during immobilization [25, 26]. This makes the opening of the lipase by other external interfaces unnecessary, and it becomes a guarantee of the immobilization of the lipases in the monomeric and open form [3]. In some cases, like lipase B from *Candida antarctica* the lid is very small and does not cover the whole active site but in some other cases like lipase LipA from *Bacillus subtilis*, there is not lid domain is present and the active-site [27–30].

Thus, the immobilization on hydrophobic supports is of interest for lipases because can promote the one-step immobilization/stabilization/purification and hyperactivation [13, 31, 32]. The improvement of stability is very important because the low stability of immobilized lipases in anhydrous hydrophobic media (for example, oils) is a drawback for the successful development of enzymatic processes in solvent-free systems [33]. Interfacial adsorption of lipases on hydrophobic supports (e.g. polyacrylic resins covered by C18 moieties), is one of the most interesting immobilization protocols in anhydrous media. The open active center of the lipases is fixed on the support surface with minimal distortion by the organic solvents. In addition to that, these immobilized derivatives were much more active than other immobilized ones [34] when working in fully anhydrous media. Perhaps the presence of a small and exact amount of water surrounding the immobilized lipase is now less necessary since the open and active form of the enzyme is already stabilized on the support surface. Moreno et al., have demonstrated to the ethanolysis of sardine oil in anhydrous hexane is 1000-fold faster when catalyzed by TLL adsorbed on hydrophobic supports as compared with other immobilized derivatives and with the commercial preparations [35].

In this work, the immobilization of two novel low-cost free lipases Eversa® Transform and Eversa® Transform 2.0 by adsorption on four different hydrophobic supports, Lewatit-DVB, Purolite-DVB, Sepabeads-C18, and Purolite-C18 are proposed. The objective of this study was to investigate the production of fatty acid ethyl (FAEE) and methyl (FAME) esters in the transesterification of sunflower oil solvent-free in an anhydrous medium.

## Experimental

### Materials

Visking Dialysis Tubing 12-1400 Da (Medicell membranes); molecular sieves 3 Å, ethyl oleate, p-nitrophenyl butyrate (p-NPB) were from Sigma Chemical Co. (St. Louis, USA). Sunflower oil high oleic 80 wt% was from Aceites del Sur-Coosur S.A. (Madrid, España). Lewatit VP OC 1600 (Lewatit DVB) was purchased from Bayer (Leverkusen, Germany). Sepabeads EC-OD (Sepabeads-C18) were kindly donated by Resindion S.R.L. Life Tech. Purolite ECR8804F Octadecyl acrylate (Purolite-C18) and Purolite Divinyl ECR1061 (Purolite-DVB) were from Purolite. Liquid lipases *Eversa® Transform* (from genetically modified *Thermomyces lanuginosus)* and *Eversa® Transform* 2.0 (from genetically modified *Aspergillus oryzae* microorganism) were generously donated by Novozymes (Denmark). Other reagents and solvents used had analytical or HPLC grades.

### Methods

#### Standard Enzymatic Activity Assay for Immobilization Monitoring

The hydrolytic activity was determined to verify the immobilization yield. This assay was performed in a spectrophotometer with a thermostatic cell and continuous magnetic stirring (500 rpm). The enzymatic activity was determined by measuring the increase in absorbance at 348 nm (ϵ= 5.150 M^−1^ cm^−1^) produced by the release of *p*-nitrophenol (pNP) in the hydrolysis of 0.5 mM p-nitrophenyl butyrate (pNPB) in 25mM sodium phosphate buffer at pH 7,0 and 25 °C. To start the hydrolysis reaction, 0.2 mL of lipase solution (blank or supernatant) or suspension was added to 2.5 mL of substrate solution (0.5 mM pNPB). The unit of enzyme activity was calculated as the amount of enzyme required to hydrolyze 1µmol of pNPB per minute (U) under the conditions described [21, 36].

#### Immobilization of Lipases on Hydrophobic Supports

The enzymatic immobilization on hydrophobic supports occurred through adsorption at a low ionic strength (5 mM) in sodium phosphate buffer at 25 ^o^C and pH 7,0 [37]. Recurrently, the suspension and supernatant were analyzed by enzymatic activity assay for pNPB hydrolysis, mentioned above, to verify the immobilization yield. The immobilized enzymes were filtered, washed with 25 mM sodium phosphate pH 7,0, and washed with distilled water. In both immobilizations, the lipase loading of the adsorbed preparations was 20 mg/mL of the carrier, with yields higher than 95 wt%, as compared to the initial lipase activity. Protein concentration was measured by the Bradford method [38].

#### Dehydration of Lipase Derivatives

For dehydration firstly hexane and acetone were dry by overnight incubation in the presence of an excess of molecular sieves 3 Å (500 g of molecular sieves per liter of solvent). After 5 g of lipase derivative was suspended in 50 mL of dry hexane with 20, 40, 60, 80, and 100% acetone at 25 °C for 3 h. Thus, the water presents in the pores of derivatives was dissolved and diluted in the dry solvents.

#### Enzymatic Synthesis of FAEE and FAME

For the synthesis of methyl and ethyl esters was addicted to batch reactor 10 wt% dehydrated immobilized lipase, 0.2 g molecular sieve (3Å), and sunflower oil:alcohol (ethyl or methyl alcohol) in different molar ratios (MR). The reaction was carried out at 40 °C and 150 rpm [15]. In the synthesis of ethyl esters were investigated the molar ratios ethanol:oil of 4:1or 3:1. In the synthesis of methyl esters were studied molar ratios methanol:oil of 11:1, 4:1 or 3:1. The molecular mass of sunflower oil was calculated according to the fatty acid composition, considering the composition of 85 wt% of oleic acid, 10 wt% of palmitic acid and 5 wt% of linoleic acid. To the kinetic study of reaction, 0.1 mL of the samples of the reaction were periodically drawn for analysis by HPLC (High performance liquid chromatography). The activity of derivates was calculated in the ethanolisys reaction in anhydrous media. The unit of enzyme activity was defined as the amount of enzyme necessary to consume 1 µmol of oleic acid per minute. The activity was expressed in µmol g^−1^ min^−1^ (U g^−1^) for immobilized derivatives.

#### Stability of Eversa Transform Immobilized on Sepabeads Support

The stability study was conducted as described by Remonatto [15]. Eversa Transform immobilized on Seapabeads was incubated with 4.1mL hexane and alcohol (ethyl or methyl alcohol) at 40 °C for 7 days. Periodically, 0.3 g of catalyst was a draw and directed for the transesterification reaction.

#### HPLC Analysis

The content of esters was determined by RP-HPLC (Spectra Physic SP 100 coupled with UV detector Spectra Physic SP 8450) using a reversed-phase column (C18-Ultrabase-C8, 150 × 4.6 mm, 5 μm). 0.1 mL of the reaction sample was diluted in 0.9 mL of hexane. In the conditions: injection volume of 10 µL, the flow rate of 1 mL/min, column temperature 40^o^C, and UV detection 215 nm. The reservoir A contained water, reservoir B contained acetonitrile, and the reservoir contained 2-propanol–hexane (5:4, v/v). Was used a 25 min ternary gradient with two linear-gradient steps: 30% A +70% B in 0 min, 100% B in 10 min, 50% B +50% C in 20 min, followed by isocratic elution with 50% B+50% C for 5 min. To calculate the yield of the esters, the retention time (RT) and the peak corresponding to the pure compound analyzed were identified (ethyl oleate RT 12.2 min). [39].

#### Cycles of Use Immobilized Lipases on Sepabeads Support

Cycles of use of immobilized lipases were conducted under the conditions: 10 wt% of immobilized lipase, MR ethanol: oil sunflower 3:1, and 0.2 g molecular sieve (3 Å), incubated at 40 °C, 150 rpm an orbital shaker for 3 h. When the reaction time was over, the derivatives were washed with hexane, and a new reaction was started. This occurred by successive cycles until the yield ester decreased considerably.

## Results and Discussion

### Immobilization of Eversa® Transform and Eversa® Transform 2.0 in Different Carriers

After evaluating the behavior of lipases Transform Eversa and Eversa Transform 2.0 immobilized in the production of esters with hexane in a previous study [15] the production without solvents was investigated.

The enzymatic transesterification of sunflower oil with ethanol in a solvent-free medium to produce FAEE using two commercial lipases, Eversa® Transform and Eversa® Transform 2.0, was evaluated in this work.

The lipases were immobilized by interfacial adsorption on four hydrophobic supports with different characteristics Sepabeads-C18, Lewatit-DVB, Purolite-C18, and Purolite-DVB. In all cases, the enzyme loading of the adsorbed preparations was 20 mg ml^− 1^ of support, with immobilization yields higher than 95 wt%, as compared to the initial enzyme activity. Table 1 shows the activity and production performance of the FAEE for each of the catalysts studied.

**Table 1:** Activity of the Eversa® Transform and Eversa® Transform 2.0 lipases immobilized on different supports, using 10 wt% immobilized lipases and MR 4:1 ethanol:oil, in 24 h of reaction

Support	Eversa Transform activity (U g^−1^)	Eversa Transform FAEE (wt%)	Eversa Transform 2.0 activity (U g^−1^)	Eversa Transform 2.0 FAEE (wt%)
Sepabeads-C18	141.3±3.5	66.5±1.2	208.3±3.7	98±2.1
Lewatit-DVB	88.61±1.4	41.7±0.5	117.9±1.9	55.5±0.8
Purolite-C18	144.7±0.5	68.5±3.2	182.8±3.6	86±1.6
Purolite-DVB	130.3±2.3	61.3±0.4	138.3±1.9	65.1±4.1

Evaluating the performance of the lipase Eversa Transform (Table 1), it appears that the reaction activity varied between 88.61 U g ^− 1^ and 144.7 U g ^− 1^ using the different supports. Also, the yield of the ester after 24 h of reaction was different for the supports, producing 41.7 wt% with the lipase immobilized in Lewatit-DVB, this being the lowest production, 61.3 wt% using the Purolite-DVB support, 66.5 wt% with Sepabeads-C18 support, and 68.5 wt% for immobilization on Purolite-C18.

The immobilized Eversa transform 2.0 lipase showed a higher reaction rate than the Eversa lipase, resulting in values ​​between 117.9 U g ^− 1^ and 208.3 U g ^− 1^. Lipase Eversa 2 also showed lower values ​​in FAEE yield and activity when immobilized in Purolite DV (65.1 wt%) and Lewatit (55.1 wt%) and better results when immobilized in Purolite C18 (86 wt%) and Sepabeds C18 (98 wt%). The lipase Eversa Transform 2.0 stood out producing higher final content of FAEE (98 wt%) and in general, it had a greater activity compared to lipase Eversa Transform.

Both lipases Eversas immobilized on hydrophobic supports showed high enzymatic activities. High activity of the immobilized enzyme may be related to the immobilization of the lipase in the open form. According to Fernandez-Lafuente [21], the immobilization of lipases on hydrophobic supports has been attributed to the interfacial activation of the lipases versus the support surface.

Four commercial resins were used as hydrophobic supports in this study. Purolite C-18 and Sepabeads C-18 are both methacrylic resin functionalized with octadecyl groups. Purolite-DVB and Lewatit DVB are methacrylic resins, non-functionalized divinylbenzene groups. The results obtained using lipases immobilized the Purolite and Seapabeads supports with octadecyl groups showed a higher FAEE content. The difference in the performance of the lipases immobilized with the different supports may be linked to the characteristic of the support itself, such as structure and properties [20]. Thus, the morphology and functional hydrophobic moieties of the support where the lipase is immobilized are determinants in the reaction.

The amount of octadecyl functional groups available at the surface of the polymer create a highly hydrophobic environment that allows for better activation of the lipase during the immobilization process [25]. The increase of octadecyl groups lead also to a decrease both in the pore volume and pore diameter size of the resin leads to a high density of immobilized enzyme available leading to higher enzyme activity [25, 40, 41].

The excellent immobilization performance of Eversa lipases in Seapabeds C18 was also observed in previous work, in which the enzymatic transesterification of sunflower oil with ethanol in hexane to produce FAEE was evaluated [15]. In this previous work, both lipases immobilized in Seapabeds also were able to recognize the three positions of the oil, thus allowing to achieve high yields close to 100 wt%.

Lipases usually are sn-1,3-regioselective due to these achieve esters yields of up to 66 wt%, here the immobilized lipases in Lewatit- DVB had FAEE yield was only 55 wt%, in this case, the immobilization in this support might have altered the selectivity of lipase however, further investigation is needed.

Due to the excellent yields obtained with both enzymes, Eversa® Transform and Eversa® Transform 2.0, immobilized in Seapabeads-C18, we chose to follow the investigation of the production of esters and the behavior of immobilized lipases only on this support.

### Effect of Alcohol in the Transesterification of Sunflower Oil Catalyzed by Sepabeads–Eversa Lipases

**Fig. 1:**
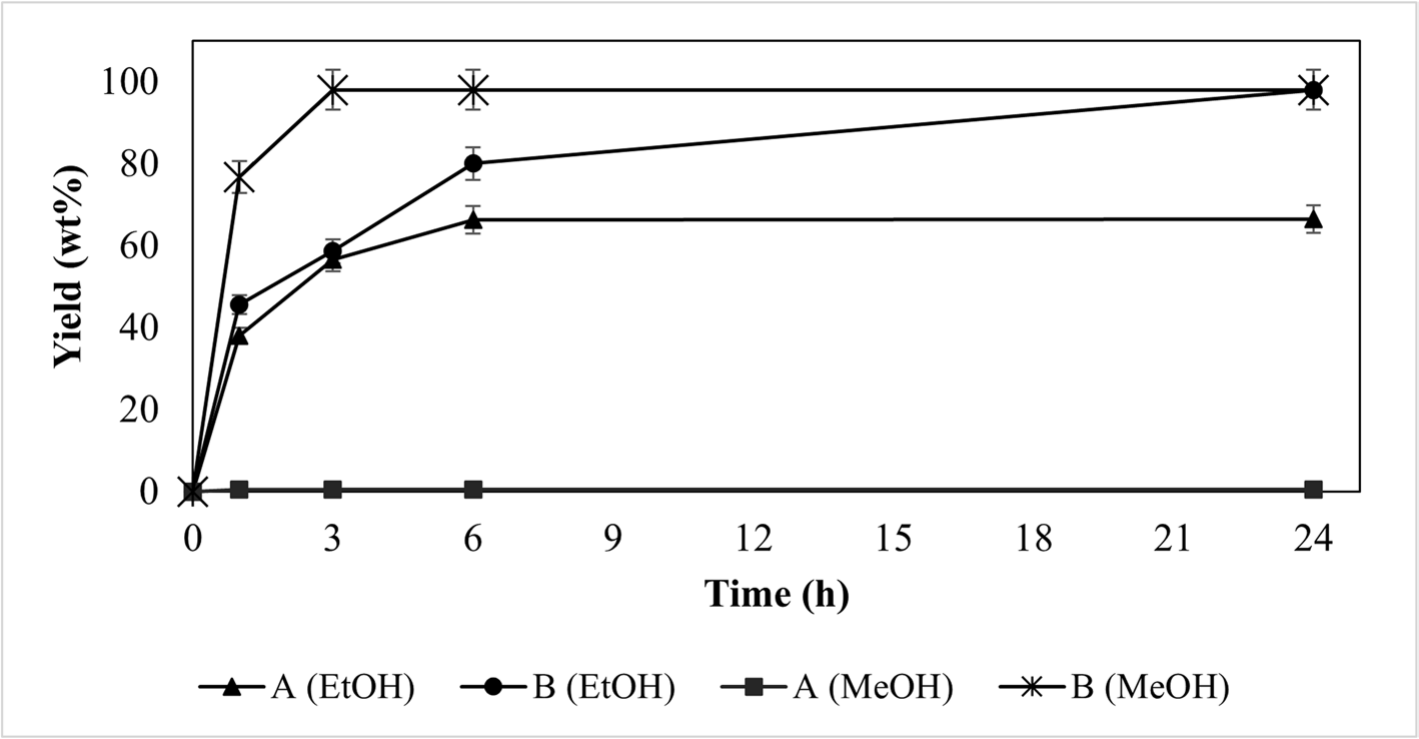
Acyl donor effect on transesterification using the Eversa® Transform (**A**) and Eversa® Transform 2.0 (**B**) lipases immobilized on Sepabeads-C18 in the conditions 10 wt% immobilized lipase, and the molar ratios (MR) of oil: ethanol of 4:1 and oil: methanol of 4:1

To evaluate and expand the application of lipases in solvent-free transesterification reactions, we studied the use of two different types of alcohols as substrates to produce esters. Thus, the study of the effects of alcohol (ethanol and methanol) on the transesterification of sunflower oil with the Eversa® Transform and Eversa® Transform 2.0 lipases can be observed in Fig. 1.

Looking the Fig. 1 the reaction that had the fastest product formation, that is higher initial velocities and catalytic activities, was lipase Eversa® Transform 2.0 using methanol as substrate. With the same immobilized lipase, but using ethyl alcohol as a substrate, an initial velocity, and the catalytic activity in the formation of esters were lower. In 3 h, using the lipase Eversa Transform 2.0 is possible to get about 98 wt% FAME, but with ethanol 98 wt% FAEE is only achieved with 24 h of reaction, this synthesis is 8 times slower.

The Eversa® Transform enzyme (Fig. 1) showed lower yields in both FAME (0.5 wt%) and FAEE (66.5 wt%) transesterification of sunflower oil in solvent-free reaction. Because of the minimal formation of FAME using the immobilized lipase Eversa® Transform, the denaturation of this enzyme may have occurred due to the excess of methanol offered in this assay.

The performance of the Eversa® Transform enzyme showed lower yields in both FAEE and especially in FAME compared to the Eversa® Transform 2.0 enzyme. The ideal molar ratio of alcohol to oil in a reaction is highly dependent on specific characteristics of lipase and the type of alcohol used for synthesis. A study conducted by Mibielli [9] using a Lipase Eversa Transform 2.0 in its liquid form for the production of biodiesel, indicated in synthesis the preference of this lipase for a higher molar ratio methanol, they using a 7.2: 1 molar ratio of methanol to residual soybean oil as ideal for production in laboratory and pilot plant biodiesel production.

### Effect of Molar Ratio Alcohol in the Transesterification of Sunflower Oil Catalyzed by Sepabeads–Eversa Lipases

To better investigate as alcohol concentration affects lipases and the formation of esters we tried to maximize the reaction using a different molar ratio of alcohol. The alternative molar ratios between oil and alcohol (methanol or ethanol) were based on previous studies [15].

**Fig. 2:**
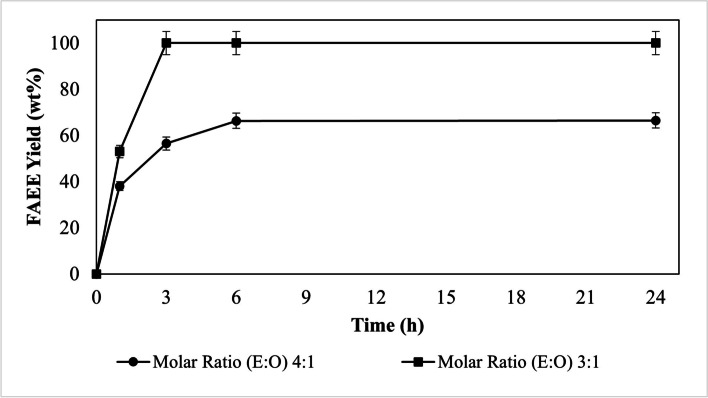
Effect of different molar ratios of alcohol on the sunflower oil transesterification reaction using Eversa® Transform immobilized on Sepabeads C-18 with ethanol using 10 wt% immobilized lipase

In Fig. 2, it can be seen that using different concentrations of ethanol it is possible to increase the reaction speed and FAEE yield for the Eversa® Transform lipase. Using the lowest molar ratio 3:1 (E: O) it is possible to achieve the total conversion of the oil to FAEE in 3 h of reaction.

However, with methanol, the behavior of Eversa Transform is completely different, even when using the lowest molar ratios of 3: 1 (M:O). In a previous study, Eversa Transform immobilized on Seapabeads-C18 was used for the transesterification of sunflower oil in an anhydrous medium using hexane as a solvent, the performance of the immobilized enzyme was higher, producing 100 wt% FAEE in 6 h and 60 wt% FAME in 24 h of reaction. In an investigation by Remonatto [8] using a Transform Eversa in its free form for the production of FAME from waste oils and methanol, esters content greater than 97 wt% were reached, however for the stability of the enzyme, the presence of water in the reaction medium was essential.

It’s possible that the stability of the Eversa Transform immobilized was affected by the absence of organic solvents or water in the reactions studied in this work. Thus, in solvent-free transesterification reactions, anhydrous medium, and the presence of methanol, the immobilized Eversa® Transform on Seapabeads-C18 was unable to develop its catalytic power, probably due to its denaturation.

**Fig. 3:**
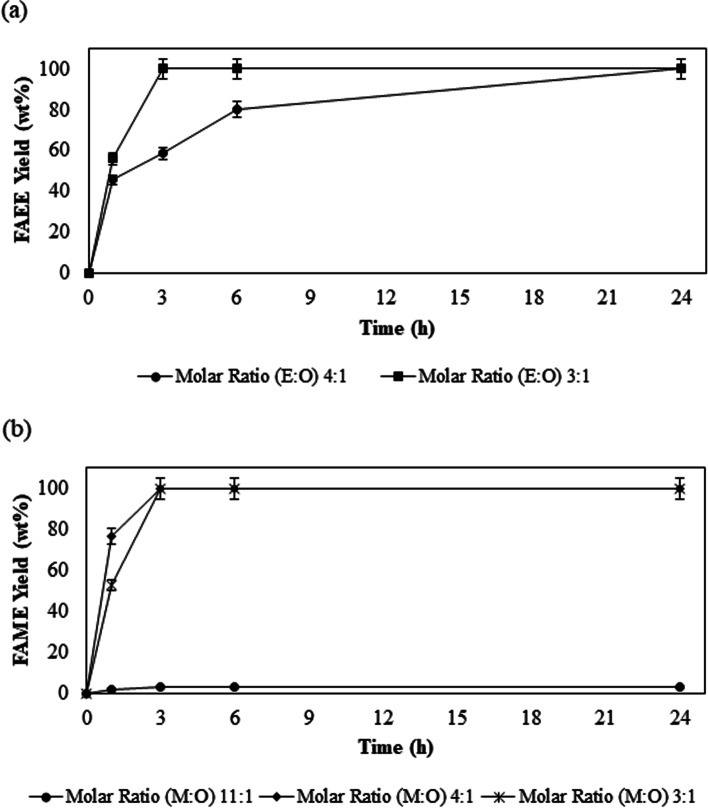
Effect of different molar ratios of alcohol on the sunflower oil transesterification reaction using Eversa® Transform 2.0 immobilized on Sepabeads C-18 with ethanol (**a**) and with methanol (**b**) using 10 wt% immobilized lipase

In Fig. 3, the decrease of the molar ratio from 4: 1 (E:O) to 3: 1 (E:O) brought benefits by increasing the reaction speed, since Eversa® Transform 2.0 achieved higher esters content (> 98 wt%) in less reaction time, only 3 h (MR 3: 1) compared if at 24 h in the reaction using 4: 1 (E:O) molar ratio. According to the literature, several studies of enzymatic esters production, using alcohol and oil as substrates, exhibit optimal molar ratio oil:alcohol 1:3 [42–47] or 1:4 [10, 11, 15, 48–51].

The high conversion in FAEE or ethyl oleate (> 98 wt%) with both lipases Eversas immobilized was obtained in a short time, in 3 h of reaction. Chiaradia [47] obtained 85 wt% ethyl oleate using the ethanol/oil molar ratio of 3:1, 7 wt% Candida Antarctica B lipase immobilized on magnetic poly (urea-urethane) nanoparticles at 50° C and stirring at 150 rpm for 4 h Ferrero [52] evaluated the production of ethyl esters from used frying oil and ethanol, using *Pseudomonas fluorescen* lipase immobilized on supports modified SBA-15 mesoporous systems, in a batch system, using the conditions, 4 wt% water, 1: 4 oil:ethanol molar ratio, 0.4 g enzyme immobilized, at 37 ° C and stirring at 180 rpm, where, after 24 h of reaction, obtained a 90 wt% FAEE yield.

In the study of the effect of methanol on the transesterification reactions with the Eversa® Transform 2.0 (Fig. 3b) was investigated novels ratios molar. The lipase had already performed well in the assay using the 4: 1 (M:O) molar ratio, with this, established two new molar ratios: a highest 11: 1 (M:O) and a lowest 3: 1 (M:O), providing for the immobilized lipase a greater range to evaluate its performance.

As observed the Fig. 3b with the highest molar ratio 11: 1 (M:O), the Eversa Transform 2.0 did not better perform, not producing relevant FAME values (<2 wt%). With the molar ratios 4: 1 (M:O) and 3: 1 (M:O) and in the reaction time of 3 h, it reached high ester content (> 98 wt%), however, the initial velocity in the reaction was lower with the molar ratio 3: 1 (M:O). Molar ratio 4: 1 (M:O) produce good results in a short reaction time (3 h), with a higher initial velocity and more importantly in a solvent-free and anhydrous medium. Facin [14] immobilized the lipase Eversa Transform 2.0 in flexible polyurethane (PU), where its necessary 24 h to convert 91 wt% FAME in the operational conditions were 2 wt% of water, 2.0 eqv. of methanol, 300 ppm of NaOH, and 500 ppm of the enzymatic cofactor.

In the research of the Eversa Transform 2.0 immobilized on Seapabeads-C18 was using for the transesterification of sunflower oil in an anhydrous medium using hexane as a solvent, the performance of the immobilized enzyme in a higher molar ratio of methanol 12:1 (M:O) was better resulting in yield 80 wt% FAME, the solvent absence affects the excellent performance this immobilized lipase. Chang [11] investigated the methanol tolerance of liquid lipase Eversa Transform 2. According to with study the enhanced methanol tolerance of the enzyme may be attributed to the gentler environment created by the water. Because this the Eversa Transform 2 has preferred for esterification reaction that can water generated from esterification of FFA.

The analysis of the stability of Eversa® Transform 2.0 immobilized on Seapabeads-C18 in the presence of ethanol and methanol was previously studied [15]. For the Eversa Transform 2.0 immobilized on Sepabeads, the stability in the presence of ethanol was greater than methanol, in ethanol, in 7 h the lipase activity remained stable at 95 wt% but after 7 h of incubation in methanol, the derivative loses more than 50 wt% of its initial activity [15].

**Fig. 4:**
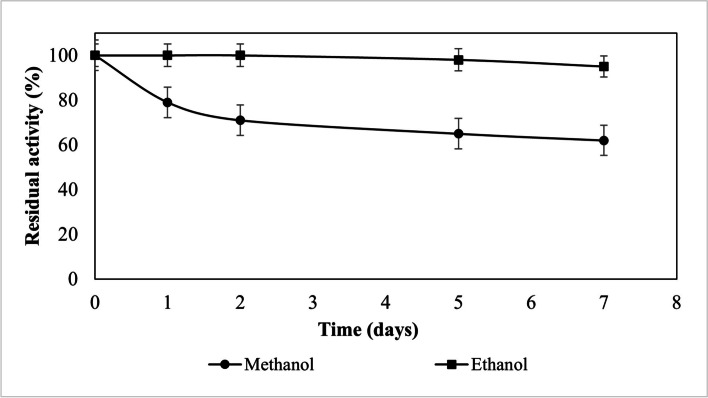
Stability in alcohol of Eversa® Transform lipase immobilized on Sepabeads-C18 on the sunflower oil transesterification reaction, using 10 wt% of immobilized lipase, 4.1 mL of hexane, and 4:1 molar ratio of ethanol/methanol:oil

Here the stability of Eversa® Transform immobilized on Seapabeads-C18 was study. The stability of Eversa Transform (Fig. 4) in the ethanol was better, in 7 days the lipase activity remained stable at 95 wt%, but with methanol, the activity reduced for 62 wt% of the original in 7 days. The answer found for stability to ethanol were similar for both Eversa lipases, but in the stability, of methanol, the performance of the Eversa® Transform 2.0 lipase was worse.

Transesterification reactions were carried out in the total absence of water and organic solvent, even so, both lipases showed good results (>98 wt%) in the production of FAEE and FAME. The solvent-free reaction requires a much higher stabilization of the immobilized biocatalyst because anhydrous oils are a very deleterious medium [33]. Therefore, strategies are needed to increase the stability of the enzyme in the Sepabeads-C18 support. With greater stability of the enzyme in the Seapabeads-C18 support, it will be possible to protect the active center of the lipase from denaturation/inactivation.

Abreu [33] investigate the *Thermomyces lanuginosus* lipase (TLL) immobilization by interfacial adsorption on octadecyl (C-18) supports. The TLL immobilized on Purolite C-18 was 20 times less stable in anhydrous oil than in anhydrous hexane. As a strategy for improving the stability was made a mild PEGylation of immobilized TLL, greatly increased its stability in hexane anhydrous fully preserving the activity after 20 days, and in anhydrous oil, the PEGylated TLL-Purolite C-18 retained 65 wt% of its initial activity after six days compared to 10 wt% of the activity retained by the unmodified biocatalyst.

As the immobilized lipases, Eversa® Transform and Eversa® Transform 2.0 are more stable to ethanol when compared to methanol. Allied to the good performance of both immobilized lipases in the reactions of ethyl oleate production, these were selected for the study of the use cycles in transesterification reactions using sunflower oil and ethyl alcohol, in an organic solvent-free system.

### Cycles of Use of the Eversa® Transform (A) Eversa® Transform 2.0 (B) lipases in the Synthesis of FAEE

The study of use cycles for both Eversa lipases immobilized on Sepabeads-C18, in solvent-free reactions, for the ethanolysis of sunflower oil, were investigated in the conditions: molar ratio of 3:1 (E:O), 10 wt% immobilized lipase at 3 h of reaction.

**Fig. 5:**
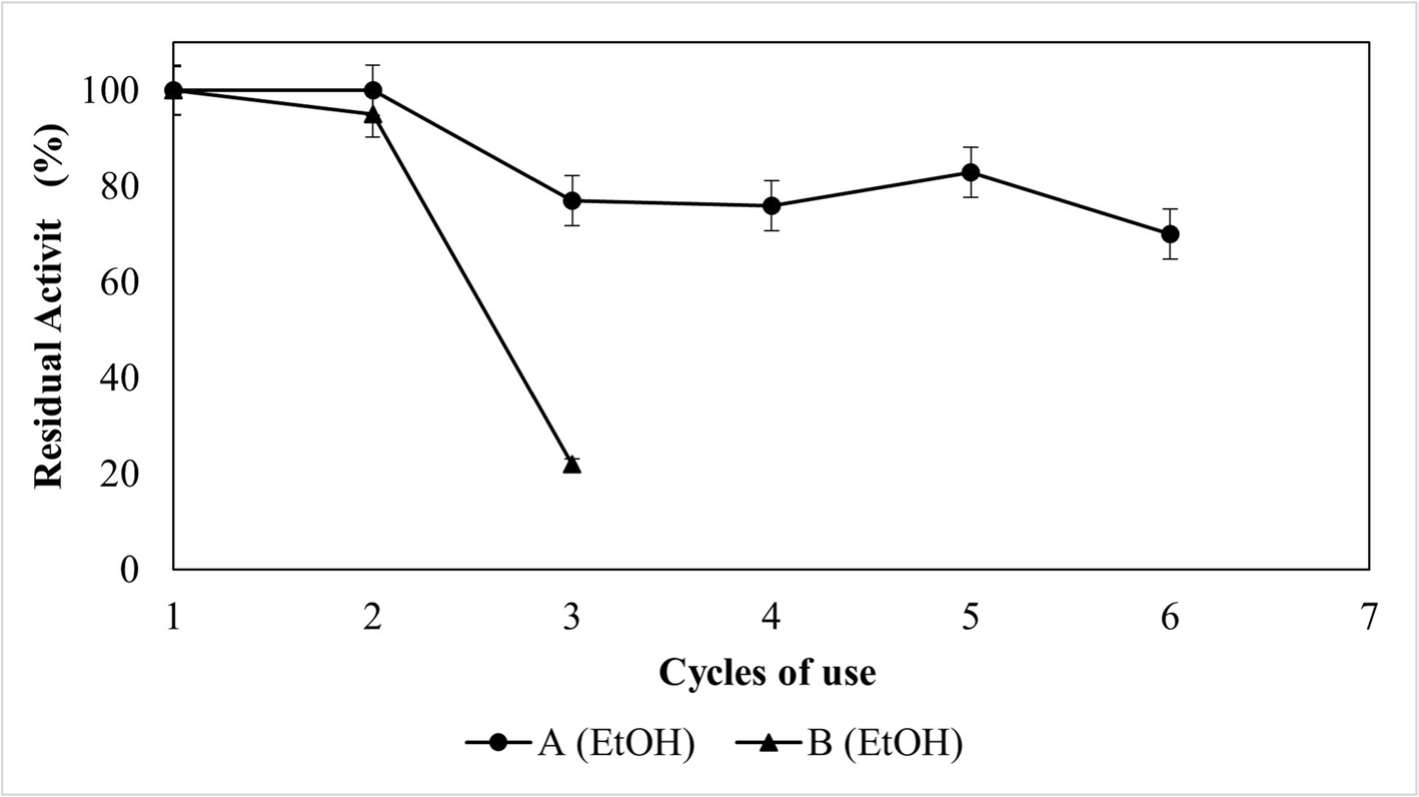
Cycles of use of the *Eversa® Transform* (**A**) and *Eversa® Transform 2.0* (**B**) lipases immobilized on Sepabeads-C18 using molar ratio ethanol: oil 3:1 and 10 wt% of immobilized lipase. The residual activity was measured by synthesis FAEE

As depicted in Fig. 5 the immobilized lipase Eversa® Transform kept the FAEE content high (> 98 wt%) for 2 cycles, after that, it presented about 75 wt% for another 3 cycles (cycles 3, 4, and 5) and 70 wt% FAEE in cycle 6. The lipase Eversa® Transform 2.0 was able to maintain a high ester content in the first (98 wt%) and second (95 wt%) cycles, however, in the third it presented less than 20 wt% ethyl esters.

In a study previous [15] the reuse of Sepabeads–Eversa® Transform 2.0 in the ethyl oleate production an anhydrous medium using hexane as a solvent, has better results, allowing the use of immobilized lipase for 4 reaction cycles. The performance of immobilized lipase in a solvent-free medium is completely different than in an organic solvent medium. As already described, the stability of the derivatives in anhydrous oil is generally less. To improve the stability of the enzymes studied here, it would be interesting to use protein engineering techniques in the soluble lipase [53, 54]. Allied to protein engineering techniques is interesting the application of post-immobilization techniques studied in other works by our research group, which investigated immobilizations for similar enzymatic processes [15, 33–35, 55, 56]. The modifications of enzymatic derivatives as the PEGylation study by Abreu [33] can be studied in future works in the Eversa Transform 2.0 lipase immobilized on Sepabeads-C18 as a strategy to improve lipase stabilization and achieve better results in its reuse in solvent-free reactions.

As a result of these results, it is evident that despite the high catalytic speed presented by the lipase Eversa® Transform 2.0 throughout the study in solvent-free reactions, an Eversa® Transform lipase is the one that presents the best performance in the transesterification reactions of sunflower oil with ethyl alcohol in a solvent-free medium. The Eversa® Transform lipase immobilized in Seapabeads-C18 to presenting high production of ethyl esters (> 98 wt%) and excellent reaction time for the production of the compounds of interest (3 h), it also presents a possibility of reusing lipases for up to 5 cycles with relevant values of FAEE.

## Conclusions

In the immobilization of soluble lipases Eversa Transform and Eversa Transform 2.0, by simple interfacial adsorption on four hydrophobic supports Purolite C18, Purolite-DVB, Lewatit-DVB, and Sepabeads-C18 support had immobilization yield was higher than 95 wt%. Immobilization by adsorption on hydrophobic supports is very easy and highly relevant. Besides, with the Sepabeads support the lipases Eversa showed high catalytic activity, representing the potential for application in reactions of synthesis of ethyl and methyl esters using sunflower oil in a system free of organic solvent. It was possible to maximize the results for both FAEE and FAME production. Immobilized Eversa® Transform is very active and fairly stable in anhydrous solvent-free medium with high percentage of ethanol. However, despite the high catalytic speed presented by Eversa® Transform 2.0 in these reactions, the lipase Eversa® Transform performed better in the transesterification reactions of sunflower oil with ethyl alcohol. Therefore, novel strategies are needed to increase the stability of the enzyme Eversa Transform 2.0 in the Sepabeads-C18 support. In the conditions the ethanol/sunflower oil molar ratio of 3:1, 10 wt% Eversa Transform immobilized on Seapabeads-C18 by interfacial adsorption, in addition to presenting high production of FAEE (> 98 wt%) in a short reaction time (3 h), this also presented the possibility of reuse of lipase for up to 6 cycles, with relevant FAEE values (> 70 wt% FAEE), contributing to the viability of the application of this lipase in bioprocesses. Eversa Transform immobilized on Seapabeads-C18 by interfacial adsorption is a promising biocatalyst for biodiesel synthesis.
